# Discriminative Multi-Stream Postfilters Based on Deep Learning for Enhancing Statistical Parametric Speech Synthesis

**DOI:** 10.3390/biomimetics6010012

**Published:** 2021-02-07

**Authors:** Marvin Coto-Jiménez

**Affiliations:** Electrical Engineering Department, University of Costa Rica, San José 11501-2060, Costa Rica; marvin.coto@ucr.ac.cr

**Keywords:** deep learning, speech synthesis, postfiltering, lstm

## Abstract

Statistical parametric speech synthesis based on Hidden Markov Models has been an important technique for the production of artificial voices, due to its ability to produce results with high intelligibility and sophisticated features such as voice conversion and accent modification with a small footprint, particularly for low-resource languages where deep learning-based techniques remain unexplored. Despite the progress, the quality of the results, mainly based on Hidden Markov Models (HMM) does not reach those of the predominant approaches, based on unit selection of speech segments of deep learning. One of the proposals to improve the quality of HMM-based speech has been incorporating postfiltering stages, which pretend to increase the quality while preserving the advantages of the process. In this paper, we present a new approach to postfiltering synthesized voices with the application of discriminative postfilters, with several long short-term memory (LSTM) deep neural networks. Our motivation stems from modeling specific mapping from synthesized to natural speech on those segments corresponding to voiced or unvoiced sounds, due to the different qualities of those sounds and how HMM-based voices can present distinct degradation on each one. The paper analyses the discriminative postfilters obtained using five voices, evaluated using three objective measures, Mel cepstral distance and subjective tests. The results indicate the advantages of the discriminative postilters in comparison with the HTS voice and the non-discriminative postfilters.

## 1. Introduction

In the field of speech synthesis, pursuing the creation of artificial voices with natural sound and flexibility, statistical parametric speech synthesis has been a hot topic for researchers for more than a decade [1,2]. The most common statistical models used are the Hidden Markov Models (HMM), modeling spectrum, duration, and pitch separately. More recently, deep learning-based speech synthesis has also been reported in several languages [3,4], and it can be considered the state-of-the-art for those languages where a large corpus of speech information is available.

For under-resourced languages or the first development of artificial speech, HMM-based speech synthesis is a technique commonly applied in many cases [5,6,7,8]. Despite the advantages of this technique for speech synthesis, some shortcomings concerning naturalness and overall quality have been mentioned in the many implementations in languages around the world, often referred to as buzzy and muffled sound [9]. The three principal factors that affect the quality of statistical parametric speech synthesis are limitations of the parametric synthesizer itself, the inadequacy of acoustic modeling, and the over-smoothing effect of parameter generation [2].

To improve the results obtained with this technique, some researchers have implemented postfilters, by adding algorithms as a final step to enhance the quality of the sound. Some algorithms implemented are deep generative architectures [10], Restricted Boltzmann Machines, and Long Short-term Memory (LSTM) [11].

In postfiltering with deep learning algorithms, a regression problem is established for transforming the synthesized features into the natural ones, determining the best-fit model for the relationship between both. This regression problem is usually one single function for a set of parameters, i.e., the spectrum information of the Mel Frequency Cepstrum Coefficients (MFCC).

It is known that the set of parameters, obtained from a database of naturally spoken utterances, comes from phoneme that has a different probability of occurrence. The probabilities have been studied, for example, in [12], where the most common phoneme of American English in the report was /ə/ with 9.96% of frequency, followed by /i/ with 9.75%. On the other hand, phonemes like /g/ and /h/ had a frequency of occurrence as low as 1.14% and 1.11% respectively.

Given that each HMM that represents phonemes in statistical parametric speech synthesis is trained separately, with a different amount of data from the database, it is straightforward to hypothesize that different distortions or shortcomings occur for the phonemes. The differences mean that a complex relationship exists between synthetic and natural data, relying on phonetic dependence.

Performing regression in data with such a complex relationship between groups has been explored by clustering data to establish simpler regression analysis for clusters. For example, in cluster-wise linear regression (CLR), the accuracy of linear regression is increased by partitioning space into subspaces, as has been successful in many fields [13].

Previous experiences with postfilters for speech synthesis have shown considerable enhancement of the speech signal without considering any clustering in applying the postfilter. In our approach, before applying the postfilters, a discriminating process is performed in order to separate voiced/unvoiced parameters, then train and implement the postfilters for each group independently.

### 1.1. Related Work

After the first published results of HMM-based speech synthesis, and the perception of its quality compared to other established techniques, the researchers began to search for new ways of modeling and reproducing the speech sound or to increase the quality of the results obtained so far. The results of the first published results were voices with high intelligibility and flexibility in most cases, but lack of naturalness in the sound.

One of the ideas presented to preserve the advantages of HMM-based speech synthesis but which increases the quality in a final stage was the postfiltering. This idea was proposed in [14], to reduce the gap between the sound of artificial speech and the natural speech [15]. The most common form of implementation of a postfilter is as a mapping function between parameters, performed with artificial neural networks. For example, in [16], the spectrum of the synthesized speech is enhanced using a mapping function estimated with Deep Belief Networks.

The improvement in the results of artificial speech relies on the capacity of the neural networks to perform the complex mapping between artificial speech and natural speech. And to overcome this complexity, some variants of the posftilters approach have been presented. For instance, a combination of postfilters, made by cascading restricted Boltzmann machines with one bi-directional associative memory was proposed in [17], to enhance the spectrum of synthesized speech.

Recurrent Neural Networks (RNN), in contrast to standard feed-forward networks for the postfiltering of synthesized speech was presented in [18]. The recurrent connections and structure of RNNs have been evidenced to better model the time dependency nature of the speech signal [15]. One of the types of RNN that has worked with better results is the LSTM and its bidirectional counterpart BLSTM. For example, in enhancing the Mel-cepstral coefficients of synthetic voices [19] and the fundamental frequency [11].

For both the complexity in the mapping function required to approximate the sound of the artificial speech to those of the natural speech and the new types of neural networks tested in close domains, the possibility of increasing the effectiveness of postfiltering is an open research question, that handles the possibility of building HMM-based artificial voices (of particular importance in low resource languages) with better quality than those of the base system.

In all the references cited previously, the mapping between artificial and natural speech is performed using the entire sequence of parameters, without considering the specific nature of such parameters. This is one of the main factors that made the mapping function so complex. In our approach, we present for the first time a discriminative postfiltering, for enhancing the synthesized speech by a group of deep learning networks trained to map the voiced or the unvoiced sounds separately.

With this approach, the postfiltering is performed in the test stage, by separating the utterances into voiced (those sounds with fundamental frequency $f_{0}>0$) and unvoiced segments (those sounds with fundamental frequency $f_{0}=0$), to enhance each one with the correspondent artificial neural networks. After the enhancing process, the segments are concatenated and the utterance resynthesized.

### 1.2. Contribution

In this paper, we extend the single postfilter approach for the enhancement of artificial speech previously presented in the literature, to a set of independent postfilters applied to subsets of phonemes defined according to its voiced/unvoiced classification.

The objective of the study is to address the following questions: (I) is a discriminating voiced/unvoiced postfilter based on LSTM capable of improving the traditional single postfilter approach for enhancing HMM-based speech synthesis? Our experimental results will affirm this question. (II) Does the discriminative postfilter allow a significant subjective preference regarding naturality? The subjective and objective test reflect this fact.

The rest of this article is organized in the following sections: In the Section 2, the Problem statement is presented. In Section 3, Long Short-term Memory neural networks are briefly described. Section 4 presents our proposed system and Section 5 gives the experimental setup. Section 6 presents the results, and finally, the conclusions are presented in Section 7.

## 2. Problem Statement

In comparison to natural speech, the trajectories of parameters in HMM-based artificial speech are smoothed, due to the statistical modeling that are performed in the training of the mathematical models [20]. This smoothing influence the perceived quality of the result.

To overcome this problem, we consider the speech parameters, $R_{Y}$, of synthetic speech as a corrupted version of the parameters, $R_{X}$, of the natural speech. In a frame-by-frame alignment of both versions of the same speech, every frame of speech is parametrized using *M* features, which can be expressed as the vector:

(1) $$\vec{c}=\left[c_{1},c_{2},\cdots ,c_{M}\right].$$

With one vector representing a frame, a whole utterance of speech produces a matrix of size M×T, where *T* is the number of frames. This matrix has the form

(2) $$\vec{R}=\left[{\vec{c}}_{1}^{⊤},{\vec{c}}_{2}^{⊤},\cdots ,{\vec{c}}_{T}^{⊤}\right]$$

With this notation, let $\vec{R_{Y}}$ and $\vec{R_{X}}$ be the matrices of the parameters extracted from the synthetic and natural speech respectively, and $\vec{R_{W}}$ the concatenation of $\vec{R_{Y}}$ and $\vec{R_{X}}$.

In deep-learning-based postfiltering, enhancing the features of the artificial voice is made by approximating a function *f* directly from the data, with the aim of mapping synthetic features to natural features, using models such as Recurrent Neural Networks. This mapping can be performed by minimizing the error function [18]:

(3) $$E\left(\vec{R_{W}}\right)=||f\left(\vec{R_{Y}};\vec{R_{W}}\right)-\vec{R_{X}}{\left|\right|}^{2}$$

In our approach, we consider the whole set of parameters of an American English voice and perform a clustering dividing the set of phonemes into two mutually exclusive clusters, corresponding to voiced or unvoiced sounds. There are two functions, $f_{21}$ and $f_{22}$ trained to map the parameters on each of this clusters to the corresponding natural parameters.

**Figure 1:**
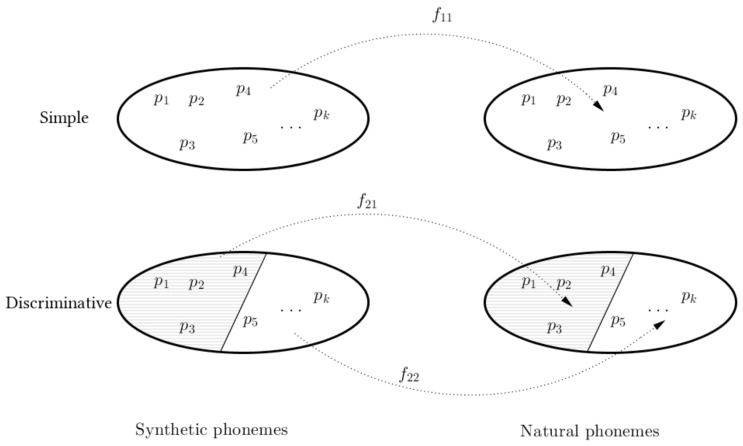
Illustration of the mapping function performed between synthetic and natural phonemes in the base system (simple) and the proposed (discriminative).

Figure 1 illustrates the discriminative clustering and the regression performed: In traditional postfiltering, a single regression function $f_{11}$ is used to map features of all synthetic phonemes to the natural phonemes. In our discriminative approach, one partition to the space of phonemes is performed, and two independent functions, $f_{21}$ and $f_{22}$ are used to map the features from cluster 1 of synthetic speech to the cluster 1 of natural speech, and the same with cluster 2.

The clustering and regression trained for each cluster are finally applied to a test set of utterances, to evaluate the enhancing obtained each level of the hierarchical clustering and determine at which level the enhancing is more successful, regarding several quality measures.

For the regression task, we chose Long Short-Term Memory Neural Networks, which have been proved successfully in several speech-related tasks, including postfiltering. The next section gives details on this kind of neural networks.

## 3. Long Short-Term Memory Neural Networks

The LSTM neural networks are an extended kind of RNN, developed with the purpose of store information in internal states of the network over long or short periods of time. The proposal was first presented in [21], and has been successfully used in speech recognition [22,23], which provides its significance in speech related tasks. But the storage and use of long-term information is potentially useful for other applications where the parameters develops depending on previous information.

In a RNN, the outputs of the network, $y=\left(y_{1},y_{2},\cdots ,y_{T}\right)$ are computed from the inputs $x=\left(x_{1},x_{2},\cdots ,x_{T}\right)$ and values from the hidden layers $h=\left(h_{1},h_{2},\cdots ,h_{T}\right)$ iterating Equations (4) and (5) from 1 to *T* [24]:

(4) $$h_{t}=H\left(W_{xh}x_{t}+W_{hh}h_{t-1}+b_{h}\right)$$

(5) $$y_{y}=W_{hy}h_{t}+b_{y}$$

where $W_{ij}$ is the weight matrix between layer *i* and *j*, $b_{k}$ is the bias vector for layer *k* and H is the activation function for hidden nodes.

The LSTM architecture and the flow of information through the network is much more complex than the traditional recurrent neural networks, given that each internal unit has several extra gates to allow the pass or the storage of information. These gates: input $i_{t}$, forget $f_{t}$, output $o_{t}$ and cell activation $c_{t}$ are implemented using the equations:

(6) $$i_{t}=\sigma \left(W_{xi}x_{t}+W_{hi}h_{t-1}+W_{ci}c_{t-1}+b_{i}\right)$$

(7) $$f_{t}=\sigma \left(W_{xf}x_{t}+W_{hf}h_{t-1}+W_{cf}c_{t-1}+b_{f}\right)$$

(8) $$c_{t}=f_{t}c_{t-1}+i_{t}\tanh\left(W_{xc}x_{t}+W_{hc}h_{t-1}+b_{c}\right)$$

(9) $$o_{t}=\sigma \left(W_{xo}x_{t}+W_{ho}h_{t-1}+W_{co}c_{t}+b_{o}\right)$$

(10) $$h_{t}=o_{t}\tanh\left(c_{t}\right)$$

where σ is the sigmoid function $f:R\to R,f\left(t\right)=\frac{1}{1+e^{-t}}$ and $W_{mn}$ are the weight matrices from each cell to the gate vector.

A detailed description of the training procedure of LSTM networks can be found in [25].

## 4. Proposed System

In our proposal, we use the HTS system to provide aligned versions of natural and synthesized speech, in order to reduce the gap between them. Given each synthesized utterance, we extract vectors of parameters in each frame, using the Ahocoder system [26]. Each vector consist of one coefficient for $f_{0}$, one coefficient for energy, and 39 Mel-frequency cestral coefficients (MFCC).

The parameters are processed independently, as proposed in previous references [11], and after the parametrization, we separate the parameters in voiced (with a value of $f_{0}>0$) and unvoiced (with a value of $f_{0}=0$ according to the Ahocoder parametrization), both in the synthesized and natural utterances. The reason of this discrimination is that voiced/unvoiced is one of the most distinctive features of the speech sounds, reflected from the source filter model of speech production [27].

**Figure 2:**
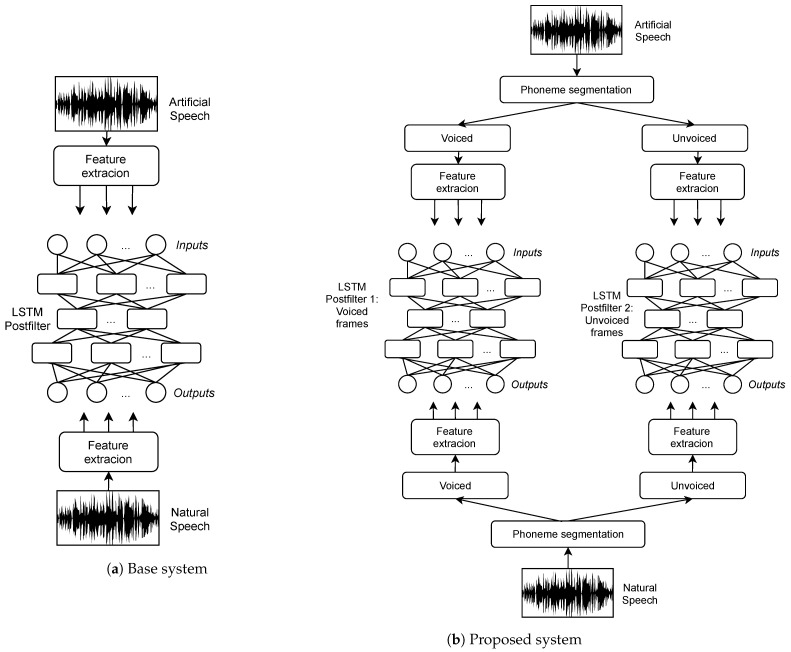
Comparison of the (**a**) base system and (**b**) the proposal. In the base system, a single postfilter is applied to the whole utterance, regardless of the nature of the individual sounds. In our proposal, the postfilter is applied selectively, by discriminating the unvoiced/voiced nature of the sounds.

The training procedure is illustrated in Figure 2, where the base systems consists in a single postfilter, whilst the proposed system perform the enhancement separately for voiced and unvoiced frames. For each group of voiced and unvoiced frames, we train a collection of LSTM networks to enhance each parameter separately, proposing three cases with collections of postfilters, describen as follows:

- In the first type of postfilter proposed (LSTM-1), a LSTM neural network with the same number of units at the input and at the output (autoencoder) is trained, with the inputs corresponding to the MFCC parameters of each frame of the HMM-based voice, and the outputs correspond to the MFCC parameters of the natural voice for the same aligned sentence.
- In the second type of postfilter, LSTM-2, the MFCC are enhanced in the same way as the previous case LSTM-1, but a new LSTM is trained to map the energy parameter from the HMM-based voice, to the energy parameter of the corresponding natural voice, also using natural MFCC features at the input and the output during training, in a particular form of auto-associative network.
- In the third type of postfilter, LSTM-3, the difference with LSTM-2 is an additional auto-associative LSTM network trained on the $f_{0}$ parameter.

This procedure was similar to those presented in [11], but with the implementation of an additional discriminative process, that allows a further improvement in the quality of newly synthesized utterances with HTS, using distinct collections of networks as a way of refining the voiced and unvoiced sounds.

**Figure 3:**
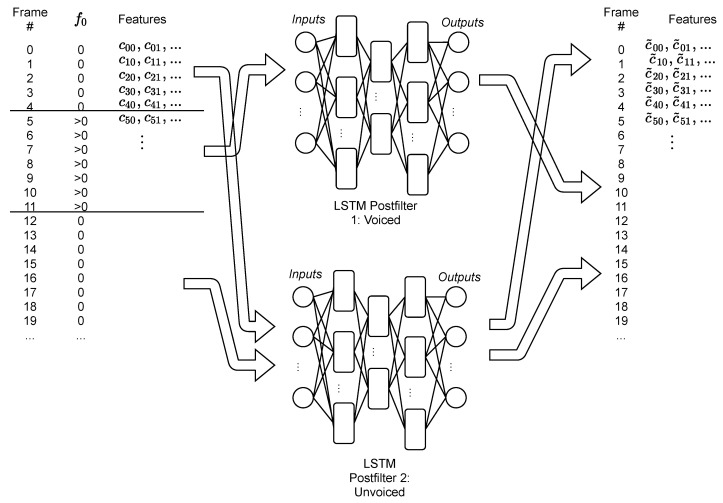
Procedure followed in the test set. Each frame is labeled with a number, and according to the value of $f_{0}$ is enhanced by one of the postfilters. After the enhancing, the frames are concatenated again and the speech is re-synthesized.

Figure 3 shows the procedure followed for the enhancing of the new utterances (test set): Each frame of the utterance is labeled with a sequential number. Then each block of voiced/unvoiced frames is separated according to the value of $f_{0}$. The blocks corresponding to voiced or unvoiced sounds are enhanced using the corresponding postfilter. The number of frames is used to properly reorganize the frames after the process and reconstruct the utterance.

## 5. Experimental Setup

### 5.1. Corpus Description

In this work, we use the CMU_Arctic database, developed by the Language Technologies Institute at Carnegie Mellon University. The database was designed to be phonetically balanced, with several US English speakers, both male and female.

Each participant recorded around 1150 utterances selected from out-of-copyright texts from Project Gutenberg. The details of this database are available in the Language Technologies Institute Tech Report CMU-LTI-03-177 [28]. Each participant is labeled using three capital letters: BDL (male), CLB (female), RMS (male), JMK (male) and SLT (female).

### 5.2. Experiments

As the general procedure for testing machine learning tasks, specially those based in neural network, the whole set of vectors or each voice was divided into training, validation, and testing sets. Table 1 shows the number of vectors in each set for each of the five voices.

The architecture of the LSTM networks were defined after a process of trail and error, with 150, 100 and 150 units in each one of the hidden layers. The final selection was taken also considering feasible training time for the total of 40 LSTM networks applied in the postfilters of the work (one for each kind of postfilter and each voice, for the discriminative and the non-discriminative cases). The training process was accelerated by a NVIDIA GPU, and took about 7 h to train each LSTM.

The following notation will be used in the results and analysis. The base system correspond to the non-discriminative approach, and the Discriminative correspond to our proposal:

- HTS: The HMM-based voice without postfiltering.
- Base-Type 1: Postfiltering of MFCCs of the HTS voice with one denoising autoencoder of LSTM network, while the $f_{0}$ and energy parameters remain the same of the HTS.
- Base Type 2: The same of Base-Type 1, with an additional auto-associative LSTM network for separately enhance the energy parameter. The $f_{0}$ parameter remain the same of HTS.
- Base Type 3: The same of Base-Type 2, with an additional auto-associative LSTM network for separately enhance the $f_{0}$ parameter.
- Discriminative-Type 1: Postfiltering of MFCCs of the HTS voice with two denoising autoencoder LSTM networks, discriminating one for voiced and one for unvoiced MFCCs. The $f_{0}$ and energy parameters remain the same of the HTS.
- Discriminative-Type 2: The same of Discriminative-Type 1, with two additional auto-associative LSTM networks: one for enhancing the energy of voiced sounds and one for the energy of the unvoiced segments of speech.
- Discriminative-Type 3: The same of Discriminative-Type 2, with one additional auto-associative LSTM network for enhancing the $f_{0}$ of the voiced sounds. The unvoiced segments of speech remain with $f_{0}=0$ and don’t need to be changed.

### 5.3. Evaluation

To assess the improvement in the quality of the synthetic voices, we use the following objective measures:

- Segmental SNR (SegSNR): Is a measure of the relation of the energy of the speech and the noise, commonly used to measure speech quality. Is implemented following the equation:
  (11) $$SegSNR=\frac{10}{N}\sum_{i=1}^{N}\log\left[\frac{\sum_{j=0}^{L-1}s^{2}\left(i,j\right)}{\sum_{j=0}^{L-1}{\left(s(i,j)-x(i,j)\right)}^{2}}\right]$$
  where x(i) is the original and $s_{i}$ the *i*th processed speech samples, *N* is the total number of samples and *L* is the frame length.
- PESQ: PESQ is a measure based on a predictive model of the subjective quality of speech. This measure is defined in ITU-T recommendation P.862.ITU. Results are reported in the interval [0.5,4.5], where 4.5 is the perfect quality of the speech, according to the reference sound (the natural recording).
  PESQ is computed with the equation:
  (12) $$PESQ=a_{0}+a_{1}D_{ind}+a_{2}A_{ind}$$
  where $a_{k}$ are adjusted to optimize the measure according to the signal distortion and overall quality.
- Weighted-slope spectral distance (WSS): This is a measure calculated in the frequency domain, comparing the slopes presented in the spectrum, calculated using the equation:
  (13) $$WSS=\frac{1}{N}\sum_{i=0}^{N}\frac{\sum_{j=1}^{K}W\left(j,i\right){\left(S_{s}\left(j,i\right)-S_{x}\left(j,i\right)\right)}^{2}}{\sum_{j=1}^{K}W\left(j,i\right)}$$
  where $S_{s}\left(j,i\right)$ and $S_{x}\left(j,i\right)$ are the slopes for the *j*th in the frame *i*. *K* is the total number of spectral bands. The weights W(j,i) are established according to the magnitude of the peaks in the spectrum.

The Mean Absolute Distance between individual MFCCs was also calculated, to measure the difference between the HTS and postfiltered voices. Finally, subjective preference score based on naturalness of the HTS and postfiltering approaches were also obtained from surveys.

## 6. Results

The results are presented in two subsection: In the first one, the performance of the algorithms within each of the discriminative and non-discriminative LSTM postfilters are presented. Statistical significance of the improvement is judged by Tukey’s HSD. All tests were performed using a significance of 0.95.

The second subsection present the results of subjective listening tests, in terms of preference scores between both approaches. Some sample audio files of the results were included as supplementary materials of this article (Audio S1–S4).

### 6.1. Objective Measures

The results for the WSS measure are shown on Table 2. In four of the five cases the best results were obtained with the discriminative postfilters, and in the fifth (BDL voice), the results of the discriminative postfilter do not differ significantly from the best.

It is also noticeable that the WSS measure for the SLT voice, the best result was for the Discriminative postfilter type 3, none of the other algorithms have a similar result. Similar results were obtained for the PESQ, as shown in Table 3 where the discriminative postfilters obtained the best results or significantly different from the best, with the SLT result of PESQ as the best, and none of the other algorithms obtained comparable results.

The result for the SegSNR_f_ results are shown on Table 4, where three of the five voices have the best result for this measure with the discriminative postfilters, and the RMS and BDL voice without a significant difference from the best.

The previous results show that the discriminative postfilters have the best results for the majority of voices, and in the rest, the results are not significantly different from the best, showing the benefits of the discriminative approach to postfiltering.

**Figure 4:**
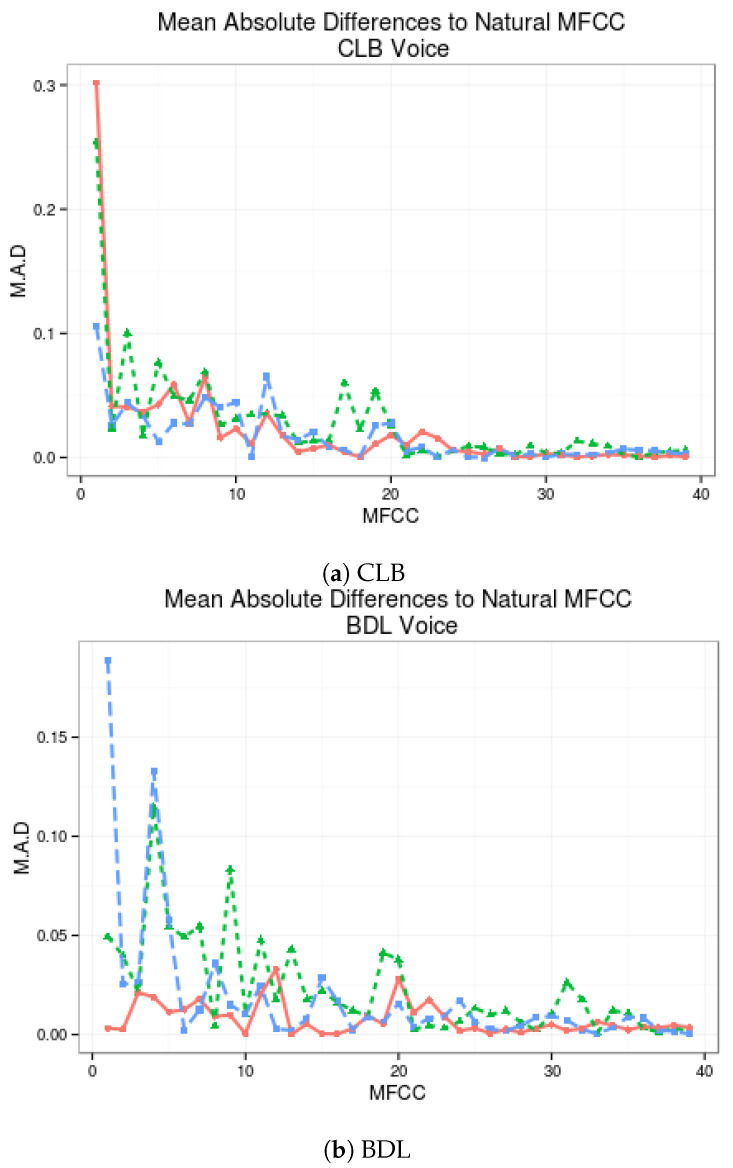

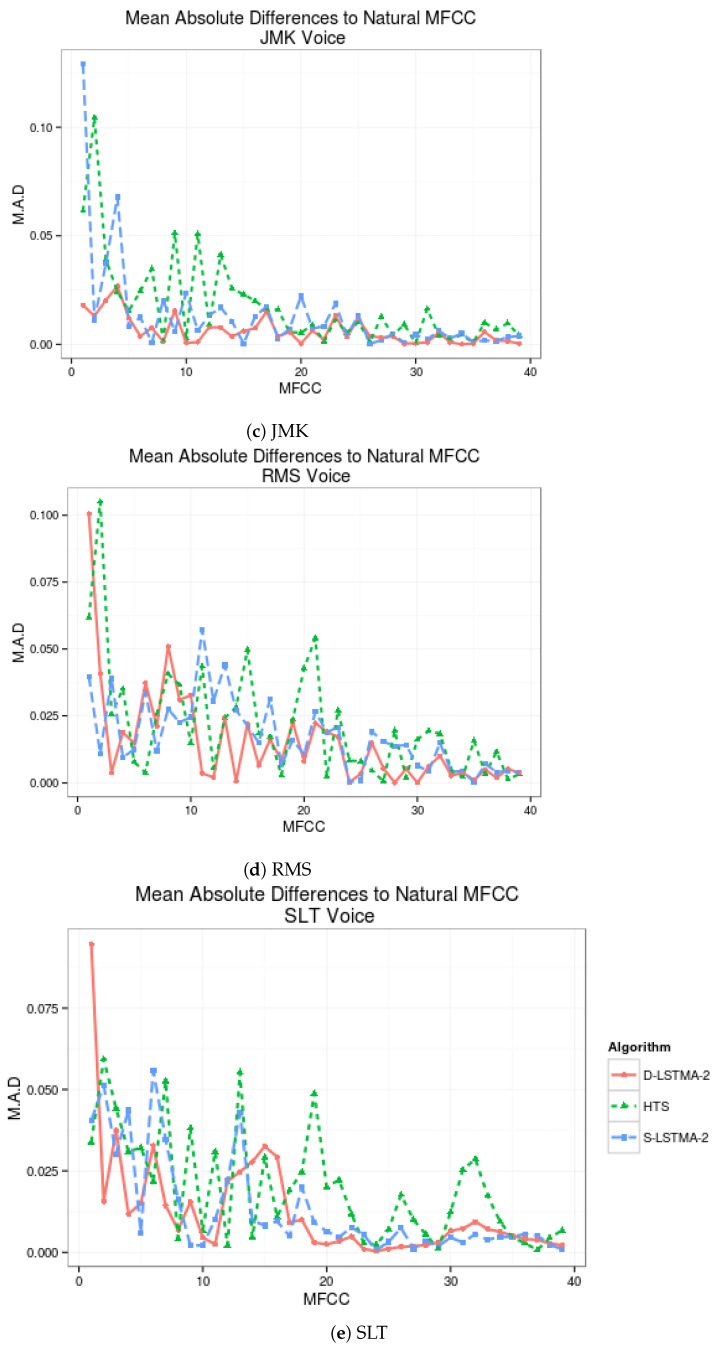
Comparison of mean differences between MFCC of each algorithm and natural voice.

On Figure 4, the Mean Absolute Distance between the MFCC of the discriminative system proposed and the standard multi-stream postfilters are presented, both compared with the MFCCs of the HTS voices without postfiltering.

Discriminative-LSTMA-1 provides better approximation to natural MFCC than regular LSTMA-1 in 20 of 39 coefficients from SLT voice (51.28%), as seen in Figure 4. Similar or greater improvements have been obtained with the other voices. The most notorious improvement comes from the JMK voice, where 29 out of 39 MFCC coefficients have been improved with the Discriminative-LSTMA-1 algorithm (74.36%) in comparison to the LSTMA-1, and the BDL voice where 24 out of 39 MFCC (61.54%) are better estimated with the Discriminative-LSTMA-1 in comparison with the LSTMA-1.

### 6.2. Statistically Significant Enhancement of the Noisy Speech Signal

In this section, we present a statistical analysis in order to determine when the results presented so far significantly enhance the HTS voice. One reason for this is the fact that a system may give the best result for a measure without significantly enhancing the HTS voice.

For the statistical analysis, we applied Tukey’s HSD test to assess significant differences between the enhanced speech signal and the HTS voices. This test gives pairwise comparisons between all results. In Table 5 the results of the test are summarized, and reported from the best case of the types described in Section 5.2. The statistical test shows the capacity of the discriminative postfilters to enhance the WSS of the HTS voice in more cases than the correspondent non-discriminative approach.

For the PESQ measures, the results show two remarkable facts: none of the postfilter enhances the PESQ measure for the CBL voice, and the Discriminative-Type was the only postfilter that obtained significant enhancement of the HTS voice for the case of SLT. The rest of the results show improvements, but not statistically significant. The significant enhancement of the SegSNR_f_ present similar results of the WSS results, where the Discriminative postfilters enhance all the voices significantly in most cases than the base case.

### 6.3. Subjective Results

The performance of the discriminative postfilters, in comparison with the non- discriminative postfilters and the HTS voices, were subjectively evaluated by perceptual tests. Twenty utterances, which were randomly selected from the testing set of all systems and voices, were evaluated according to preference tests participated by 60 subjects through an online system. All subjects are native American English speakers, both male and female, with ages between 20 and 50 years old.

**Figure 5:**
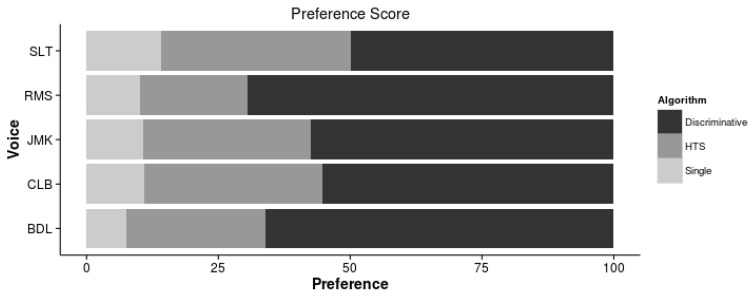
Preferece score for the HTS voices and the non-discriminative (single) and discriminative postfilters.

The preference scores are shown in Figure 5. It shows that the speech enhanced by the Discriminative postfilters is significantly preferred than the best HTS and the non-discriminative postfilters for all voices, with the most notorious differences in the RMS and BDL voices.

## 7. Conclusions

In this paper, the proposal to use discriminative postfilters to enhance the quality of artificial voices produced with statistical parametric techniques based on HMM was analyzed. The postfilters applied are based on LSTM neural networks, previously presented in the literature for their applicability in improving speech signals.

The discriminative approach of postfiltering refers to the distinction of voiced and unvoiced segments, and the consequent application of specific postfilter to each, in contrast to a single postfilter for all speech segments, as is done in the base case. The assumption for applying this discriminative approach is that the nature of voiced and unvoiced sounds differs sufficiently to treat their improvement separately.

The advantages of the proposal were verified using three objective measurements. The significant improvements were verified in comparison to the quality of the artificial voice. And also in contrast to the base case where a single postfilter is applied to the whole sentence. The improvement was also verified with subjective evaluations by a group of listeners, who indicate their preference for the sound quality of the voices processed with discriminative postfilters.

Therefore, with the results of this work, there is evidence to support the advantages of enhancing specific speech segments, produced with HMMs, instead of complete speech sentences. In the instante of mapping between complete sentences, postfilters based on LSTM neural networks must learn more complex mapping functions, contemplating mapping functions between sounds that differ much or little between natural and artificial speech.

The discrimination of sounds to enhance the quality of speech represents an advantage that could be analyzed further with more specific types of sounds; for example fricatives, plosives, liquids, or other linguistic categories. Following this path, the postfilters can be trained to provide more specific mappings and produce more significant improvements in the artificial voices.

## Figures and Tables

**Table 1:** Amount of data (vectors) available for each voice in the databases.

Database	Total	Train	Validation	Test
BDL	676,554	473,588	135,311	67,655
SLT	677,970	474,579	135,594	67,797
CLB	769,161	538,413	153,832	76,916
RMS	793,067	555,147	158,613	79,307

**Table 2:** WSS Results for the hierarchical clustering levels. The lower values represent better results. * indicates the best result. In bold are the results which are not significantly different from the best, according to Tukey’s HSD test.

Voice	HTS	Base-Type			Discriminative-Type		
		1	2	3	1	2	3
SLT	46.30	42.78	43.21	69.54	42.18	41.97	33.84 *
RMS	38.30	**32.39**	**32.54**	38.62	30.76 *	**31.39**	**31.45**
JMK	35.26	**31.69**	**31.45**	35.65	30.50 *	**31.18**	**32.10**
CLB	37.20	**34.96**	**34.94**	36.92	**32.55**	32.23 *	36.61
BDL	41.71	**37.20**	37.09 *	41.59	**37.82**	**37.60**	**38.72**

**Table 3:** PESQ Results for the hierarchical clustering levels. The higher values represent better results. * indicates the best result. In bold are the results which are not significantly different from the best, according to Tukey’s HSD test.

Voice	HTS	Base-Type			Discriminative-Type		
		1	2	3	1	2	3
SLT	1.0	1.0	1.0	0.6	1.0	1.0	1.3 *
RMS	1.5	1.6 *	**1.5**	**1.4**	1.6 *	**1.4**	**1.4**
JMK	1.3	1.4 *	1.4 *	**1.2**	**1.3**	**1.2**	**1.2**
CLB	1.3	1.2 *	1.2 *	**1.1**	1.2 *	**1.1**	**1.0**
BDL	1.4	1.4 *	1.4 *	1.1	1.4 *	1.4 *	**1.3**

**Table 4:** SegSNR_f_ Results for the hierarchical clustering levels. The higher values represent better results. * indicates is the best result. In bold are the results which are not significantly different from the best, according to Tukey’s HSD test.

Voice	HTS	Base-Type			Discriminative-Type		
		1	2	3	1	2	3
SLT	0.5	1.2	1.7	0.3	1.5	1.8	2.8 *
RMS	1.4	**2.4**	2.5 *	1.4	**2.2**	**2.0**	1.8
JMK	1.7	**1.9**	1.1	0.8	**2.0**	2.1 *	**2.0**
CLB	2.4	2.7	2.2	2.4	3.4 *	**3.1**	**2.8**
BDL	0.5	**1.4**	1.5 *	0.7	**1.3**	**1.3**	**1.2**

**Table 5:** Tukey’s test results. Ticks indicate a significant enhancement of the artificial speech, and ns means an improvement but not statistically significant. And empty space represent that no improvement were measured.

Voice	Base-Type			Discriminative-Type		
	WSS	PESQ	SegSNR_f_	WSS	PESQ	SegSNR_f_
SLT	ns	ns	✓	✓	✓	✓
RMS	✓	ns	✓	✓	ns	✓
JMK	✓	ns	ns	✓	ns	ns
CLB	ns		ns	✓		✓
BDL	✓	ns	✓		ns	ns

## Supplementary Materials

The following are available online at https://www.mdpi.com/2313-7673/6/1/12/s1, Audio S1: Artificial speech, Audio S2: Artificial Speech with Discriminative Postfiltering, Audio S3: Artificial Speech, Audio S4: Artificial Speech with Discriminative Postfiltering.

## Abbreviations

The following abbreviations are used in this manuscript:

BAM	Bidirectional Associative Memory
CMU	Carnegie Mellon University
DBN	Deep Belief Network
DNN	Deep Neural Network
$f_{0}$	Fundamental frequency
GPU	Graphics Processing Unit
HMM	Hidden Markov Models
HTS	H-Triple-S: HMM-based Speech Synthesis System
LSTM	Long Short-term Memory
MFCC	Mel-Frequency Cepstral Coefficients
PESQ	Perceptual Evaluation of Speech Quality
RBM	Restricted Boltzmann Machine
RNN	Recurrent Neural Network
SegSNR	Segmental Signal-to-noise Ratio
SNR	Signa-to-noise Ratio
WSS	Weighted-slope Spectral Distance
